# Retraction: Effects of intramedullary nailing and internal fixation plates on postoperative wound infection and pain in patients with distal tibia fractures: A meta‐analysis

**DOI:** 10.1111/iwj.70020

**Published:** 2024-08-09

## Abstract

Kou F, Li T. Effects of intramedullary nailing and internal fixation plates on postoperative wound infection and pain in patients with distal tibia fractures: a meta‐analysis. *Int Wound J*. 2023; 21(3): e14479. doi: 10.1111/iwj.14479

The above article, published online on 20 November 2023, in Wiley Online Library (http://onlinelibrary.wiley.com/), has been retracted by agreement between the journal Editor in Chief, Professor Keith Harding; and John Wiley & Sons, Ltd. It came to the publisher's attention from a third party that a number of articles shared concerning similarities in format and structure. Following an investigation by the publisher, the retraction of this article has been agreed on because the peer review and publishing process for this article were found to have been manipulated. The authors did not respond to our notice of retraction.

Kou F, Li T. Effects of intramedullary nailing and internal fixation plates on postoperative wound infection and pain in patients with distal tibia fractures: a meta‐analysis. *Int Wound J*. 2023; 21(3): e14479. doi: 10.1111/iwj.14479

The above article, published online on 20 November 2023, in Wiley Online Library (http://onlinelibrary.wiley.com/), has been retracted by agreement between the journal Editor in Chief, Professor Keith Harding; and John Wiley & Sons, Ltd. It came to the publisher's attention from a third party that a number of articles shared concerning similarities in format and structure. Following an investigation by the publisher, the retraction of this article has been agreed on because the peer review and publishing process for this article were found to have been manipulated. The authors did not respond to our notice of retraction.

